# Molecular Dynamics Investigation of Lipid-Specific Interactions with a Fusion Peptide

**DOI:** 10.3390/biom14030285

**Published:** 2024-02-27

**Authors:** William T. Heller

**Affiliations:** Neutron Scattering Division, Oak Ridge National Laboratory, Oak Ridge, TN 37831, USA; hellerwt@ornl.gov; Tel.: +1-865-241-0093

**Keywords:** HIV-1, viral fusion peptide, molecular dynamics simulations

## Abstract

The HIV-1 fusion peptide, which is a short hydrophobic peptide from the gp41 coat glycoprotein that participates in the infection of a cell, interacts with model lipid bilayer membranes in a concentration-dependent manner. The interaction of the peptide with the bilayer also strongly depends on the lipid composition. Here, molecular dynamics simulations were performed to investigate lipid-specific interactions that arise shortly after the binding of a less-fusogenic variant of the HIV-1 fusion peptide to a lipid bilayer composed of a mixture of dimyristoyl phosphatidylcholine and dimyristoyl phosphatidylglycerol. The impact of peptide concentration was also studied. An improved understanding was gained of the lipid-specific interactions experienced by the FP. New insight was also gained into how the peptide concentration changes these interactions.

## 1. Introduction

The HIV-1 virus relies on a small peptide, a short segment at the N-terminus of the gp41 coat glycoprotein, to fuse with the cellular membrane to start the infection process [1]. This portion of the gp41 coat protein is called the fusion peptide (FP). The gp41 protein forms a heterodimer with gp120 that assembles into a trimer in the virus [2]. The conformation of the FP depends on both its sequence and the environment to which it is exposed. It has been observed to be either α-helical [3,4] or a mix of α-helix and random coil [5] within the larger, isolated coat glycoprotein structure. It has also been observed to have a disordered and extended conformation by crystallography within the complex [6]. When bound to antibodies, the conformation adopted can be α-helical [7,8], extended [7,9], or β-sheet [8] depending on the specific antibody to which it is bound. Mutations that make the FP, which has the amino acid sequence AVGIGALFLGFLGAAGSTMGARS, less hydrophobic, such as by replacing hydrophobic amino acids with polar ones, result in a less infectious virus [10,11,12,13]. These studies support the important role that the FP plays in infection.

The interactions of the isolated FP and mutations of it with model lipid bilayer membranes have also been the focus of considerable study. An α-helical conformation of the peptide is generally considered to be the inactive state of the peptide [14,15,16,17]. This conformation is found when the peptide concentration is low or when the bilayer is made solely of zwitterionic lipids [14,15,16,17,18,19]. The conformation of the FP that is associated with fusion primarily has a β-sheet structure [14,15,16,17]. This conformation is found at higher peptide concentrations or when anionic lipids are part of the bilayer [14,15,16,18,19,20,21]. However, the α-helical conformation can also be seen at relatively high peptide concentrations when the bilayer contains lipids with unsaturated acyl chains [21,22,23,24,25]. The sensitivity of the peptide conformation and function to the bilayer composition raises many questions about the interactions between the peptide and the lipids.

A previous study into the lipid-dependent conformations of a less fusogenic variant of the HIV-1 FP that has the amino acid sequence RKGIGALFLGFLGAAGSTMKR and is referred to as gp41rk revealed that the peptide preferentially interacted with specific phospholipids in model lipid bilayer membranes [21]. The peptide was studied in three different lipid mixtures, namely 7:3 DMPC:DMPG, 7:3 POPC:POPG and (7:3 DMPC:DMPG):(7:3 POPC:POPG). Only the acyl chain composition differed. Experiments revealed that the conformation of the peptide, which was present at a P/L of 1/50, and its impact on the lipid bilayer with the quaternary composition was more similar to what was observed in the 7:3 POPC:POPG lipid mixture than in the 7:3 DMPC:DMPG mixture. MD simulations revealed that the peptide in the quaternary mixture preferentially associated with lipids with one monounsaturated acyl chain and charged lipids, namely POPG, POPC and DMPG, at the expense of DMPC. The observed preferential association provided an explanation for the experimental results observed [21].

Previously obtained evidence of lipid-specific interactions [21] leads to questions about whether lipid-specific interactions exist as the peptide concentration increases. Here, an MD simulation study of gp41rk interacting with bilayers composed of DMPC and DMPG is presented, again in a 7:3 molar ratio. Previous experiments found that the peptide conformation depends on its concentration in this lipid mixture, but fusion was not observed [19,21], making it well-suited for additional study by MD. All-atom simulations were performed at peptide-to-lipid ratios of 1/200, 1/100 and 1/50. In this concentration range, the peptide conformation was experimentally observed to transition from an α-helix-containing structure to one possessing a β-sheet structure [18,19]. The simulations provide new data about lipid-specific interactions taking place when FPs interact with lipid bilayer membranes in the period of time shortly after binding takes place. The results also provide new insight into how the lipid–protein interactions of the FP change with peptide concentration.

## 2. Materials and Methods

### 2.1. General Simulation Information

All molecular dynamics simulations were performed using GROMACS [26]. Different versions were employed depending on the task at hand. When used on a local desktop computer for preparatory runs performed before the production runs described below, version 2021.4 was used. Analyses of the simulation trajectories were performed on a local desktop computer as well, used the 2021.4 version for all analyses, and employed the built-in tools with one exception. The 2023.1 version has a built-in tool for determining the secondary structures of the peptides present in the simulation, *dssp*, that is not present in the 2021.4 version of GROMACS. All production runs of the simulations described below were performed using the CADES computational resources at Oak Ridge National Laboratory and employed the 2019.5 version of GROMACS. The force field parameters used for the lipids were the “Stockholm lipids” set [27,28,29,30], which are an expanded set of the parameters developed for CHARMM [31]. The AMBER99SB-ILDN force field [32], which is compatible with the “Stockholm lipids” force field, was employed for the simulations containing peptides.

The starting configuration for the peptide-free bilayer containing a 7:3 molar mixture of DMPC and DMPG was taken from the results of a previous study [21]. The starting peptide-free bilayer had 35 waters per lipid, which can be considered fully hydrated. The starting structure also contained sodium counterions to make the system charge neutral. The I-TASSER server [33,34] was used to produce a starting structure for gp41rk. Of the various structures produced, an α-helical one was selected as the starting structure for the simulations because the peptide adopts a helix-containing structure when bound at low concentrations [18]. The desired number of copies of the peptide were placed in close proximity to the lipid bilayer to promote rapid binding using an updated version of software developed in-house for a previous study [21]. The software picks a random orientation and position within the simulation box for the peptide while only allowing positions and orientations that do not cause collisions with lipid molecules or other peptides in the box. A candidate structure was visually inspected to ensure that the axis of the peptide or peptides were roughly parallel to the bilayer surface, the peptide or peptides were near to the bilayer surface, and that no two copies of the peptides were in direct contact. When two gp41rk peptides were present, both were associated with the same leaflet of the bilayer. Two gp41rk peptides were associated with each leaflet of the bilayer when four copies of the peptide were added to the starting structure because adding four peptides to one side of the bilayer was deemed to be likely to cause too high of a strain on the structure. Sodium ions were removed from the peptide-containing starting configurations to make the systems charge-neutral.

A brief description of the peptides in the starting structures follows. In the initial structure for the GP411 simulation, the minimum distances from the atoms in the gp41rk amino acids to a lipid atom ranged from 1.8 Å to 9.2 Å. The minimum distances between acids in the amino acids of the gp41rk and lipid atoms ranged from 5.9 Å to 15.3 Å and 4.9 Å to 14.4 Å for the GP412 initial structure. The minimum distance between the peptides in the starting structure was 5.7 Å, so they were not in close contact. For the GP414 initial structure, the minimum distances from an amino acid atom to a lipid atom were 1.8 Å to 10.2 Å, 1.8 Å to 12.1 Å, 1.8 Å to 10.2 Å and 1.8 Å to 9.4 Å for the four gp41rk peptides present. In all cases, the helical axes were roughly parallel to the bilayer surface. The closest distances between the pairs of peptides associated with each bilayer leaflet were 7.6 Å and 14.0 Å, so neither pair of peptides was in contact. No peptides were in contact across the periodic boundary conditions, either.

### 2.2. Common Simulation Parameters

Many commonly used parameters were used throughout the various simulations performed, except, as noted, for specific cases detailed within the simulation protocols described below. The leap-frog integrator was employed with a time step of 2 fs. All electrostatic calculations used the particle mesh Ewald (PME) approach [35]. Both the electrostatic calculations and the van der Waals calculations used a distance cut-off of 1.2 nm. In the peptide-free simulations, two temperature coupling groups were employed. The lipids were one group, while the water and ions were the second coupling group. A third coupling group containing the copies of gp41rk was also used because the peptide or peptides were expected to transition from primarily interacting with the water to having increased interaction with the lipids. During the short NVT (number–volume–temperature) simulations performed, velocity rescaling [36] with a time constant of 0.1 ps was used to maintain the temperature. A Nosé–Hoover thermostat [37,38] maintained the temperature at 37 °C (310 K) for all NPT (number–pressure–temperature) simulations. The time constant of the thermostat was set to 0.5 ps. Parrinello–Rahman semi-isotropic pressure coupling [39] was used in the NPT simulations to maintain a pressure of 1 bar. The time constant for the pressure coupling was set 5 ps.

### 2.3. Simulation Protocols

The GP410, GP411 and GP412 simulations utilized three steps, although the first two were not required for GP410 because the starting state was taken from a previous study [21]. The first step was an energy minimization step that employed the steepest descent minimization algorithm. A step size of 0.01 nm was used. A target maximum force of 250 kJ/mol/nm was used for GP411, GP412 and GP414. Up to 50,000 steps in the minimization process were allowed, but no system required that many steps before the minimization process was complete. Each system was then simulated for 100 ps in the NVT ensemble. At this point, the simulation protocol for GP414 deviated from the other simulations performed. For GP410, GP411 and GP412, a short equilibration run (1 ns for GP410 and 10 ns for GP411 and GP412) was performed and checked to verify that the system was behaving reasonably. In the case of GP414, early tests showed that some copies of the peptide did not fully associate with the leaflet of the bilayer that they were placed next to in the starting configuration. These copies of gp41rk would then extend across the periodic boundary conditions such that the free end would associate with the other leaflet of the bilayer. Therefore, the NVT run was followed by a 1 ns simulation run in which the peptides were pulled along the *z*-direction toward the center of mass of the lipids to ensure that the peptides had an opportunity to associate with the head group region of the bilayer along as much of the length of the sequence as possible. Force was applied using the umbrella potential with a force constant of 100 kJ/mol/nm^2^. Then, the force was turned off, and a 10 ns NPT equilibration run was performed for the GP414 system.

Finally, NPT production simulations were performed for the total times shown in Table 1. The total simulation times were broken into 100, 200, 400 or 500 ns blocks. Breaking the overall simulation into smaller segments was necessary for working within the restrictions of the computational resources available for the study. It was also possible to easily inspect the trajectories at various intervals. In all cases, the final production run block was 200 ns in length, and it was used for the majority of the analyses presented unless otherwise indicated. The longer time afforded to GP414 was deemed necessary due to the increased complexity of the peptide–peptide and peptide–bilayer interactions that could take place in the system.

### 2.4. Simulation Trajectory Analysis

The analysis performed on all simulation trajectories that is reported in the Section 3 was restricted to the final 200 ns of the simulations performed, except as noted. Simulation quality checks of the total energy, temperature, pressure and box dimensions were performed using the *energy* tool, although the results are not presented herein. Distances and contacts between various groups were calculated using *mindist*, and the distances and contacts were monitored over the entire period of the production runs plus during the first, short NPT equilibration run. The mass density profiles along the direction normal to the plane of the bilayer were calculated using the *density* tool for the system, the lipid phosphate groups and the peptide or peptides. The phosphate peaks were fit using Gaussian functions for their positions and widths, and the reported uncertainties were those reported by the fitting. Radial distribution functions (RDFs), g(r), were calculated for the $C_{\alpha }$ atoms of gp41rk relative to the phosphorous atoms of the two kinds of lipids in the simulations. The lateral RDFs, being in the plane of the bilayer (i.e., the xy-plane), were calculated for each leaflet of the bilayer. The secondary structures of all copies of the peptides included in the simulations were calculated using the *dssp* tool implemented in the 2023.1 version of GROMACS [26], which implements the DSSP algorithm developed previously [40,41]. The results of *dssp* were categorized into helices, β-structures, bends, turns and “other” structures using software developed in-house in Python. Additional software, also developed in-house in Python, was used to calculate the area-per-lipid, $A_{L}$, and to produce histograms of the results. This software leveraged the *LiPyphilic* software package [42] for performing the calculations, which made it possible to determine the area of each lipid and thus the average $A_{L}$ for DMPC and DMPG. *LiPyphilic* leverages the *MDAnalysis* [43,44] software package for handling the trajectory data. It also uses the *freud* [45] software package for the $A_{L}$ calculations.

## 3. Results

### 3.1. Peptide-Free Simulation (GP410)

**Figure 1:**
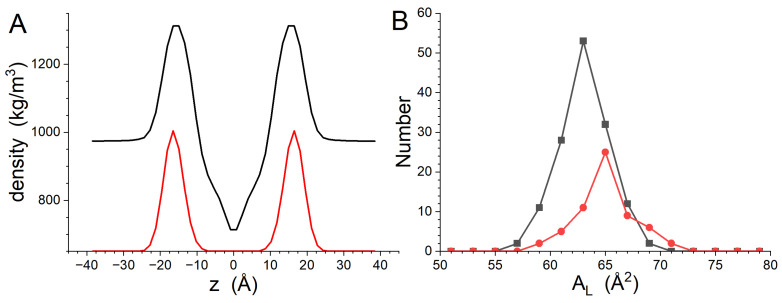
Transbilayer density profiles calculated from the GP410 simulation results (**A**). The profile for the entire system is black, while the profile calculated from the phosphate groups is red. The phosphate group density profile has a constant baseline of 600 kg/m^3^ added to it for presentation purposes. (**B**) Histograms of the time-averaged $A_{L}$ for DMPC (black) and DMPG (red).

The GP410 simulations provided the data needed to understand the impact of the peptide on the structure of the bilayer. The transbilayer density profile of the system as well as the distribution of the phosphate groups calculated from the final 200 ns of the trajectory are shown in Figure 1A. The $D_{ptp}$ of the bilayer obtained by fitting the lipid phosphate group distributions with Gaussian functions is 32.8 ± 0.1 Å, and the peak widths are 5.4 ± 0.1 Å. The uncertainty in $D_{ptp}$ was estimated from the fitting of the phosphate group distributions. Distributions of the time-averaged $A_{L}$ for DMPC and DMPG are shown in Figure 1B. The distributions are roughly symmetric about the peak. The average $A_{L}$ and standard deviation for DMPC is 63.1 ± 2.3 Å^2^, while that of DMPG is 64.9 ± 2.5 Å^2^. The experimentally determined $A_{L}$ for DMPC is 61.6 ± 1.0 Å^2^ at the same temperature [46] and 66.2 ± 1.2 Å^2^ for DMPG at 37 °C [47]. The simulation results are consistent with previously published experimental results obtained for pure lipids to within one standard deviation.

### 3.2. Simulation with One gp41rk Peptide (GP411)

**Figure 2:**
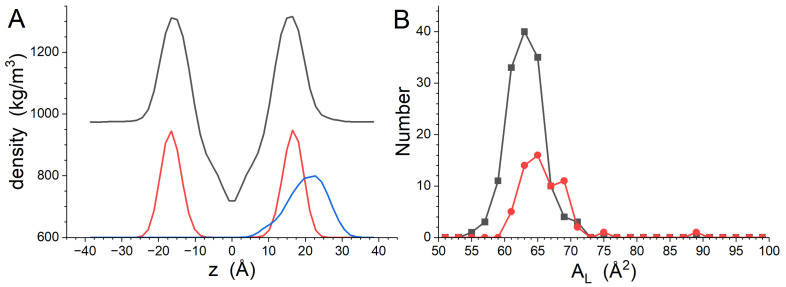
(**A**) Transbilayer density profiles calculated from the GP411 simulation results. The profile for the entire system is black, the profile calculated from the phosphate groups is red, and the density profile calculated from the gp41rk peptide is blue. The phosphate and peptide density profiles have a constant baseline of 600 kg/m^3^ added to them for presentation purposes, and the peptide density profile has been scaled by a factor of 5 for clarity. (**B**) Histograms of the time-averaged $A_{L}$ for DMPC (black) and DMPG (red).

Figure 2A shows the transbilayer density distributions of the system, the phosphate groups and the peptide. The impact of the peptide on the structure is slight, which is not surprising in light of the low peptide concentration. The $D_{ptp}$ determined from Gaussian function fitting of the the lipid phosphate group distributions is 33.3 ± 0.1 Å, which is larger than the $D_{ptp}$ of the GP410 simulation. The width of the phosphate distributions increased slightly: from 5.4 ± 0.1 Å to 5.6 ± 0.1 Å. However, it is reasonable to conclude that the difference between this result and the GP410 $D_{ptp}$ is significant. The average $A_{L}$ of DMPC is 63.2 ± 2.8 Å^2^, while it is 66.1 ± 4.1 Å^2^ for DMPG. The $A_{L}$ values of both lipids in the GP411 simulation remain within a standard deviation of the values seen in the GP410 simulation, but the impact of the peptide can be seen in the $A_{L}$ histograms in Figure 2B, particularly DMPG, which has a population of larger $A_{L}$ values than seen in the GP410 DMPG $A_{L}$ histogram.

**Figure 3:**
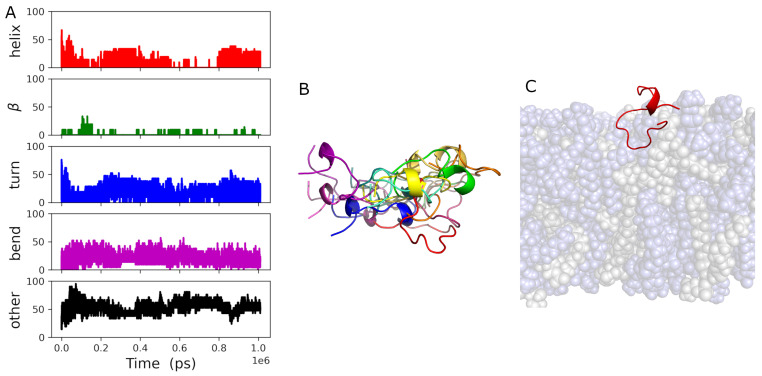
(**A**) Secondary structure percentages of the gp41rk during the GP411 simulation, including the short NPT equilibration run (see Section 2.3), that were determined as described in Section 2.4. (**B**) Examples of the gp41rk structure taken from the ensemble of structures present during the final 200 ns of the GP411 simulation. The intensity of the color indicates distance from the viewer. (**C**) The final gp41rk structure is shown with the lipid bilayer in a semitransparent representation that shows the depth of penetration towards the center of the bilayer. DMPC is light blue, while DMPG is light grey.

The peptide adsorbed to the lipid bilayer shortly after the start of the simulation and remained associated with it throughout the trajectory, including during the short NPT equilibration run, according to the results of *mindist* [26] presented in Figure S1. The density distribution for the peptide (Figure 2A) demonstrates that it is interacting more with the external surface of the bilayer than it is with the hydrocarbon core of it. Only a relatively small portion of the peptide density distribution extends below the phosphate group *z*-position during the final 200 ns presented here. The peptide conformation over the entire simulation time is shown in Figure 3A. The peptide structure is dynamic, with the secondary structure content varying over time. The secondary structure of gp41rk in the simulation has considerable turn and bend content. Previous experimental work found a mix of helix and random coil structures at P/L = 1/200 in the same mixture of lipids [19]. The variability of the structure over time can also be seen in Figure 3B, and its relationship with the hydrocarbon core of the bilayer is visible in Figure 3C. The peptide sits within the lipid headgroup region of the bilayer rather than penetrating into the hydrocarbon core, consistent with Figure 2A, and it is not greatly restricted in its lateral movement. Possible causes for the differences here and in the GP412 and GP414 results are addressed in the Section 4.

**Figure 4:**
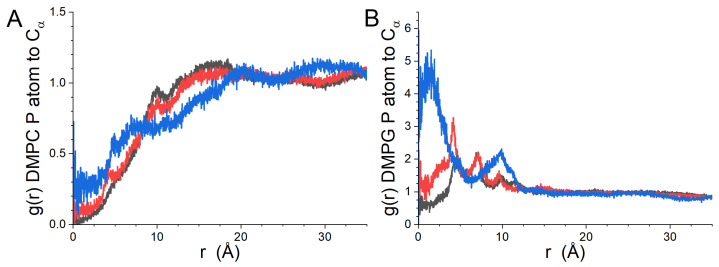
(**A**) GP410 lateral RDFs between DMPC P atoms in the leaflet of the bilayer to which the peptide is bound and gp41rk hydrophobic residues (black), polar residues (red) and positive residues (blue). (**B**) GP410 lateral RDFs between DMPG P atoms in the leaflet of the bilayer to which the peptide is bound and gp41rk hydrophobic residues (black), polar residues (red) and positive residues (blue).

The lateral RDFs between the different kinds of residues (hydrophobic, polar and positive) of gp41rk and the lipids in the leaflet of the bilayer to which it is bound are presented in Figure 4, while the RDFs for the lipids of the leaflet to which it is not bound are presented in Figure S2. The difference in the relationship between the peptide and the two lipids (Figure 4) is striking. DMPC is largely absent from the immediate vicinity of the peptide (Figure 4A). In contrast, DMPG is strongly correlated with the positively charged residues of gp41rk, and strong correlation peaks can also be seen in the RDFs for the hydrophobic and polar amino acid residues of the peptide (Figure 4B). Strong lipid–peptide relationships are not seen in the lateral RDFs for either lipid species in the opposite leaflet of the bilayer (Figure S2).

### 3.3. Simulation with Two gp41rk Peptides (GP412)

**Figure 5:**
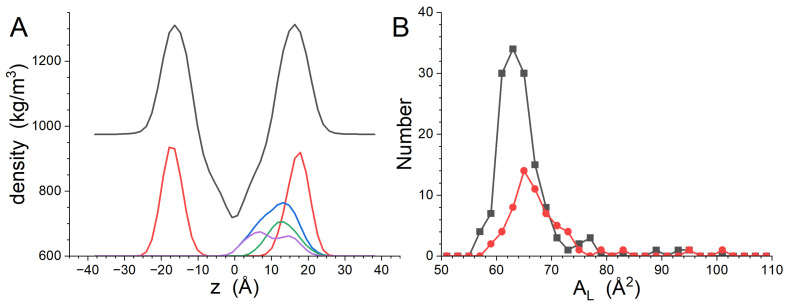
(**A**) Transbilayer density profiles calculated from the GP412 simulation results. The profile for the entire system is black, while the profile calculated from the phosphate groups is red. The density profile for all gp41rk atoms is blue, gp41rk 1 is green, and gp41rk 2 is magenta. The phosphate group and peptide density profiles have a constant baseline of 600 kg/m^3^ added to them for presentation purposes. The gp41rk density profiles have also been scaled by a factor of 2 for presentation purposes. (**B**) Histograms of the time-averaged $A_{L}$ for DMPC (black) and DMPG (red).

The transbilayer density profiles for the system, the phosphate groups and the peptides are presented in Figure 5A. The peptides have clearly impacted the structure of the bilayer, particularly the leaflet of it to which the peptides are bound. The $D_{ptp}$ determined by fitting the phosphate density distributions with Gaussian functions is 34.4 ± 0.1 Å, which is considerably thicker than the $D_{ptp}$ found for GP410 and GP411. The widths of the phosphate peaks are 5.7 ± 0.1 Å for the left peak (no peptide) and 6.1 ± 0.1 Å for the right peak (with peptide), revealing the impact of the peptide. The peptides are further toward the center of the bilayer than was seen in the GP411 simulation, with the peaks in the density distributions being below the peak of the phosphate group density distribution. Histograms of the time-averaged $A_{L}$ are shown in Figure 5B. Like the $A_{L}$ in the GP411 simulation, both DMPC and DMPG have examples of lipids with higher $A_{L}$ values than were observed in the GP410 simulation: being up to 101 Å^2^ for both DMPC and DMPG. There are more lipid molecules with high $A_{L}$ values than were found in the GP411 simulation. The average $A_{L}$ of DMPC is 64.8 ± 5.6 Å^2^, and it is 67.8 ± 7.2 Å^2^ for DMPG. Both the averages and standard deviations reflect a population of lipids with higher $A_{L}$ values. The simultaneous increase to both average $A_{L}$ and $D_{ptp}$ appears to be counterintuitive. However, the $A_{L}$ values of DMPC are larger in one leaflet than in the other, while the $A_{L}$ values of DMPG display the opposite behavior, producing the net result that the overall average $A_{L}$ of both leaflets is the same. The larger number of DMPC molecules with smaller $A_{L}$ values results in that bilayer having a phosphate density position peak that is larger than that of the other leaflet.

**Figure 6:**
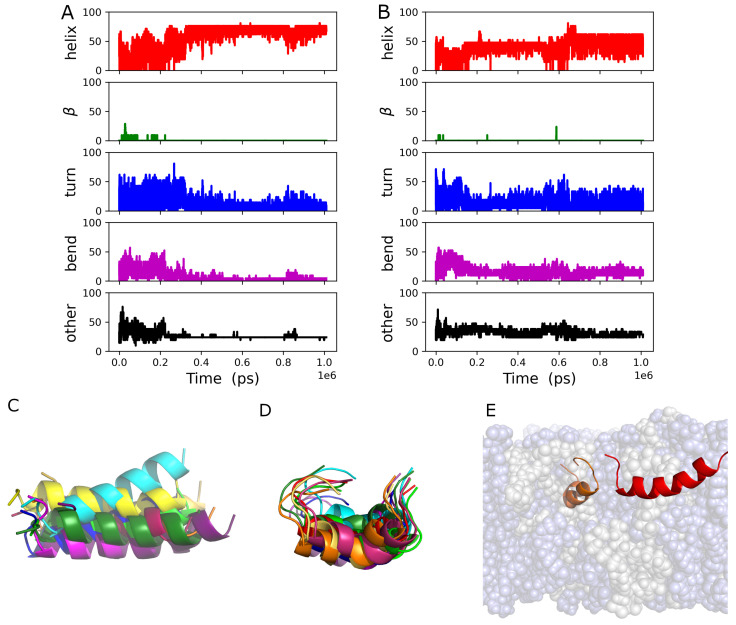
Secondary structure percentages of (**A**) gp41rk 1 and (**B**) gp41rk 2 during the GP412 simulation, including the short NPT equilibration run (see Section 2.3), that were determined as described in Section 2.4. Examples of the (**C**) gp41rk 1 and (**D**) gp41rk 2 structures taken from the ensemble of structures present during the final 200 ns of the GP411 simulation. The intensity of the color indicates distance from the viewer. (**E**) The final gp41rk structures are shown with the lipid bilayer in a semitransparent representation that shows the depth of penetration towards the center of the bilayer. DMPC is light blue, while DMPG is light grey; gp41rk 1 is red, and gp41rk 2 is orange.

As can be seen in the results of the *mindist* analysis [26] presented in Figure S3, both peptides associated with the bilayer very soon after the simulation began. The peptides did not spend a great deal of time in contact with each other based on the peptide–peptide *mindist* analysis [26], which is presented in Figure S4. The secondary structures of both peptides contained a considerable fraction of α-helix, as can be seen in Figure 6A,B, in contrast to the single peptide in the GP411 simulation. There is essentially no β-sheet structure present in the secondary structures of either peptide. The secondary structures of the two peptides are similar in the final 200 ns of the simulation. Neither peptide structure is static over the course of the simulation, much like the peptide in the GP411 simulation. Ensembles of example structures for gp41rk 1 and gp41rk 2 are shown in Figure 6C,D. While the highly helical secondary structure of gp41rk 1 is relatively stable, it moves around more within the bilayer structure than gp41rk 2. The final structure in the GP412 simulation is show in Figure 6E and reveals how deeply the two peptides penetrated into the hydrocarbon core of the bilayer.

**Figure 7:**
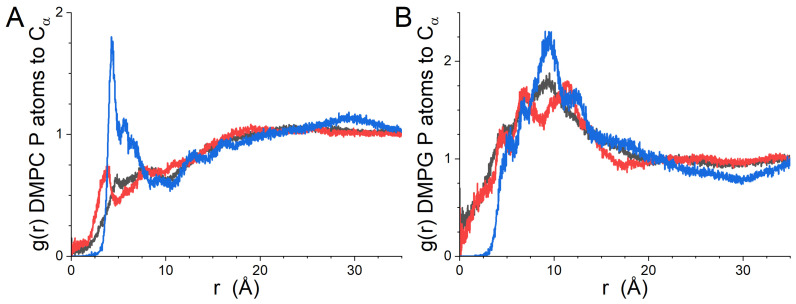
(**A**) Lateral RDFs from the GP412 simulation results between DMPC P atoms in the leaflet of the bilayer to which the peptides are bound and gp41rk hydrophobic residues (black), polar residues (red) and positive residues (blue). (**B**) Lateral RDFs from the GP412 simulation results between DMPG P atoms in the leaflet of the bilayer to which the peptides are bound and gp41rk hydrophobic residues (black), polar residues (red) and positive residues (blue).

The lateral RDFs calculated for the leaflet of the bilayer with peptides bound in the GP412 simulation results are shown in Figure 7, while the lateral RDFs of the opposing leaflet of the bilayer are shown in Figure S5. The results for the three different kinds of amino acid residues shown in Figure 7 are different than those calculated from the GP411 trajectory (Figure 4). The most striking differences are seen in the results for DMPG (Figure 7B) and for the positive residues relative to the P atoms of DMPC (Figure 7A, blue curve). The increased depth of the peptides in the bilayer relative to the one gp41rk in the GP411 simulation has partially alleviated the exclusion of DMPC from the vicinity of the hydrophobic and polar residues of the peptide seen in the GP411 simulation (Figure 4A), which is reasonable because the acyl chains of DMPC and DMPG to which the peptides are more exposed are identical. The clear lack of any Cα atoms of positively charged residues within ~2.5 Å of a lipid phosphorous atom suggests that the charged amino acid residues are consistently positioned at roughly the same height in the bilayer as the phosphate groups, which would exclude them from being closer than the contact radius in the lateral RDFs. However, the correlation of these residues with DMPC is significantly stronger than for the hydrophobic or polar residues, while the lateral RDFs for DMPG display peaks of comparable strength for all kinds of amino acids between 2.5 Å and 15.0 Å, indicating that the peptide interacts preferentially with the charged lipid. The phosphate group carries the negative charge on DMPC and DMPG, making the close relationship with the positively charged residues of the peptide electrostatically favorable. As was seen in the GP411 simulation, the peptides in the GP412 simulation are not having a strong effect on the lateral organization of the lipids in the opposite leaflet of the bilayer (Figure S5).

### 3.4. Simulation with Four gp41rk Peptides (GP414)

**Figure 8:**
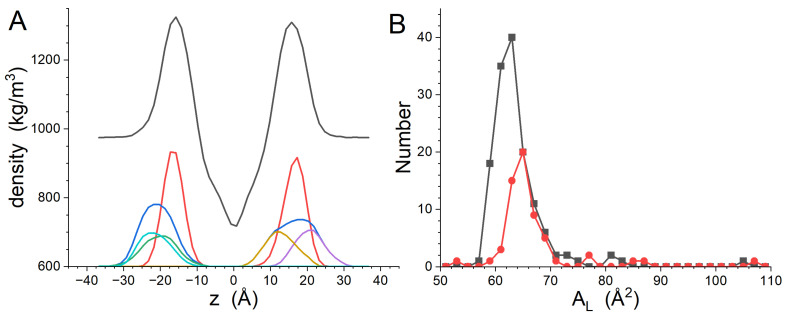
Transbilayer density profiles calculated from the GP414 simulation results (**A**). The profile for the entire system is black, while the profile calculated from the phosphate groups is red. The density profile for all gp41rk atoms is blue, gp41rk 1 is green, gp41rk 2 is magenta, gp41rk 3 is gold and gp41rk 4 is cyan. The phosphate group and peptide density profiles have a constant baseline of 600 kg/m^3^ added for presentation purposes. The gp41rk density profiles have also been scaled by a factor of 2 for presentation purposes. (**B**) Histograms of the time-averaged $A_{L}$ for DMPC (black) and DMPG (red).

The transbilayer density distributions for the system, the phosphate groups and the peptides are shown in Figure 8, while the $A_{L}$ histograms are presented in Figure 8B. Here, the $D_{ptp}$ determined from the phosphate density distribution is 33.2 ± 0.1 Å, which is smaller than the GP412 result and is consistent with the GP411 $D_{ptp}$. The widths of the left and right phosphate distributions are 5.6 ± 0.1 Å and 6.0 ± 0.1 Å, respectively. The four gp41rk density distributions reveal that only one of the copies of the peptide (gp41rk 3) was located such that the peak of its distribution is closer to the center of the bilayer than the phosphate group distribution of the leaflet with which it is associated. The $A_{L}$ values are 63.8 ± 5.5 Å^2^ and 66.5 ± 7.5 Å^2^ for DMPC and DMPG, respectively, and the histograms shown in Figure 8B again display populations of lipids with considerably larger $A_{L}$ values than those of the lipids within the main peaks of the histograms. Unlike the $A_{L}$ values in the GP412 simulation, there is no imbalance between the DMPC and DMPG $A_{L}$ in each leaflet, reflecting the rough balance of peptides bound to the bilayer.

**Figure 9:**
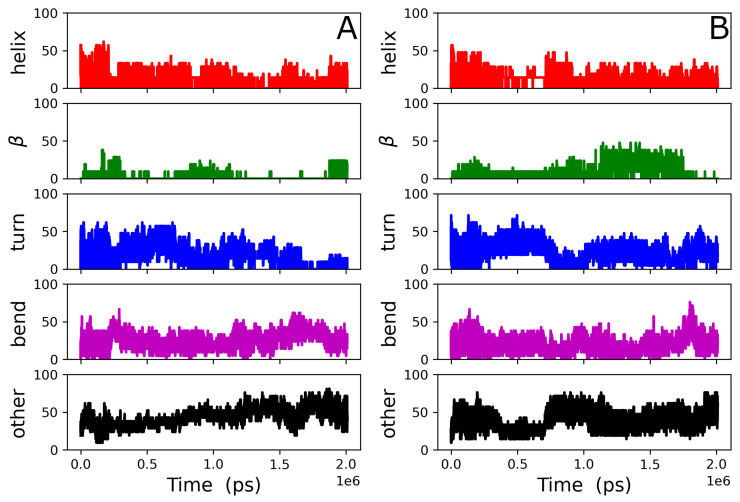

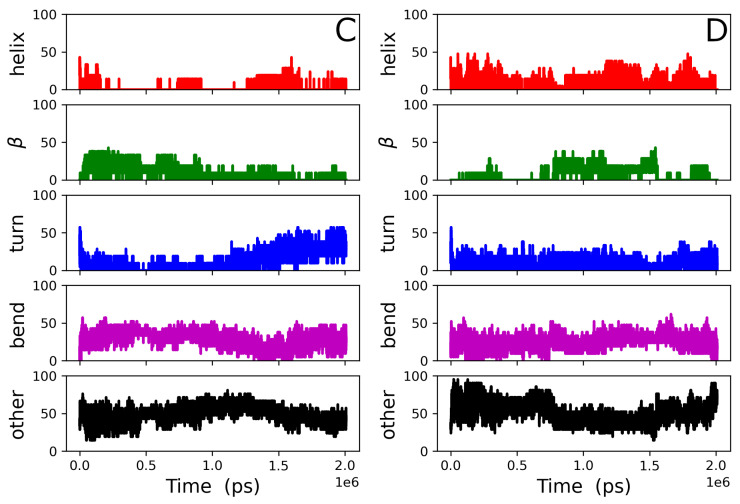
Secondary structure percentages of (**A**) gp41rk 1, (**B**) gp41rk 2, (**C**) gp41rk 3 and (**D**) gp41rk 4 during the GP414 simulation, including the short NPT equilibration run (see Section 2.3), that were determined as described in Section 2.4.

All four of the peptides remained associated with the bilayer after the applied force (Section 2.3) was turned off, which can be seen in the results of the *mindist* analysis [26] presented in Figure S6. Peptides 1 and 4 associated with one leaflet of the bilayer, while peptides 2 and 3 associated with the other. Each pair of peptides associated relative early in the trajectory according to the peptide–peptide *mindist* analysis [26], which is presented in Figure S7. However, the gp41rk 2–gp41rk 3 pair dissociated at ~100 ns before re-associating around ~200 ns and remaining so. The secondary structures of all four peptides, shown in Figure 9, were dynamic throughout the 2 μs trajectory. The secondary structures found in the simulation were mixtures of the different kinds of secondary structures. It is interesting to note that the one copy of gp41rk (gp41rk 3) that penetrated as deep into the bilayer as the peptides in the GP412 simulation did not adopt a strongly α-helical conformation.

**Figure 10:**
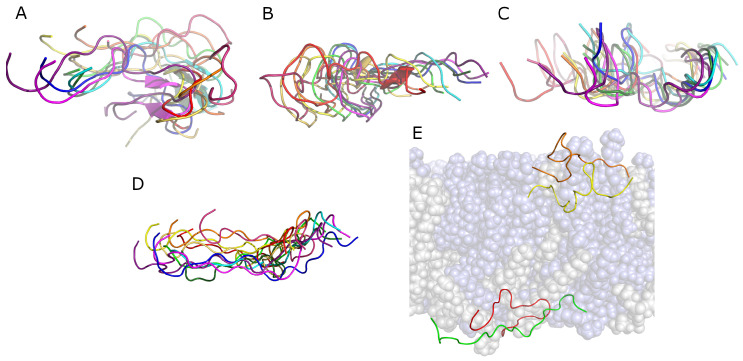
Representative structures from the GP414 simulation for (**A**) gp41rk 1, (**B**) gp41rk 2 (**C**) gp41rk 3 and (**D**) gp41rk 4 during the GP414 simulation. The intensity of the color indicates distance from the viewer. Not all of the panels are to the same scale, which was dictated by the overall spatial extent of the structure displayed. (**E**) The final gp41rk structures are shown with the lipid bilayer in a semitransparent representation that shows the depth of penetration towards the center of the bilayer. DMPC is light blue, while DMPG is light grey; gp41rk 1 is red, gp41rk 2 is orange, gp41rk 3 is yellow, and gp41rk4 is green.

Structures taken from the final 200 ns of the MD trajectory are shown in Figure 10. All four copies of the peptide are structurally dynamic, with only gp41rk 4 (Figure 10D) being more spatially constrained within the bilayer than the other three peptides. The amount of movement within the plane of the bilayer is not entirely expected because the peptides are associated into relatively stable pairs in each leaflet. Figure 10E shows the final structures of the four peptides with the lipid bilayer. Of the four peptides, only one (gp41rk 3) penetrates significantly into the hydrocarbon core of the bilayer. However, it does not sit at a depth consistent with the peptides in the GP412 simulation (Figure 6E).

**Figure 11:**
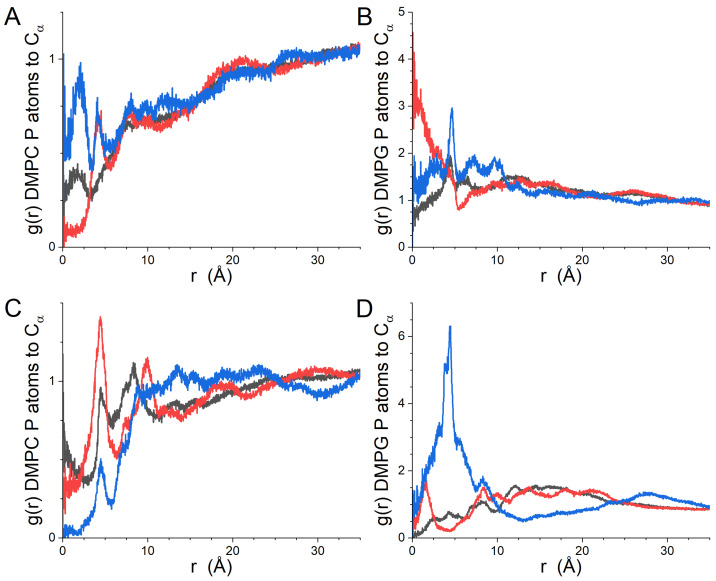
Lateral RDFs between the $C_{\alpha }$ atoms of the gp41rk pairs bound to the leaflets of the bilayer and P atoms of the lipids in the same leaflet of the bilayer as the peptides: (**A**) gp41rk 1 and 4 pair to the DMPC P atoms; (**B**) gp41rk 1 and 4 pair to the DMPG P atoms; (**C**) gp41rk 2 and 3 pair to the DMPC P atoms; (**D**) gpr41rk 2 and 3 pair to the DMPG P atoms. In all four panels, the lateral RDFs for the gp41rk hydrophobic residues are black, those for the polar residues are red, and the blue curves are the lateral RDFs calculated for the positive residues.

The lateral RDFs between the $C_{\alpha }$ atoms of the peptide pairs and the phosphorous atoms of DMPC and DMPG are shown in Figure 11. Individual peptide RDFs were not calculated because the peptides in each leaflet were associated, nor were the lateral RDFs for the opposing leaflets of the bilayer calculated because the other peptide pair was present in it. The peptides preferentially interacted with DMPG rather than DMPC, as was observed in the GP411 and GP412 simulations, but the effect is not as strong as was seen at lower peptide concentrations. The curves also display considerably more peaks than the results from the other simulations. In light of the progression of the growth of features in the RDFs with increasing peptide content, in spite of the length of the simulations, it is reasonable to conclude that the increased lateral ordering in the bilayer results from the peptide–lipid interactions rather than the length of the trajectory. Interestingly, the gp41rk 1–gp41rk 4 pair polar residues have a stronger local association with DMPG than the positively charged residues. The same is not true for the gp41rk 2–gp41rk 3 pair.

## 4. Discussion

The preferential association of gp41rk with charged lipids seen in the simulations here, and previously seen in a more compositionally complex bilayer [21], supports the importance that charged lipids play in fusion by the HIV-1 FP [14,15,16,18,19]. The results presented here show a clear affinity of DMPG for the vicinity of gp41rk associated with the bilayer, even though higher peptide concentrations reduce the effect. Electrostatic attraction between DMPG and gp41rk is one reason for the observed interactions. However, the slightly larger $A_{L}$ of DMPG compared to DMPC seen in the simulations, which is consistent with experiments [46,47], and the slightly smaller head group of DMPG [48,49] suggest a second cause. The association of peptides with lipid bilayers, such as in the surface-adsorbed states seen here, can create stresses in the bilayer due to deformation, which increases the free energy of the system [50,51]. The changes to $A_{L}$ distributions (Figure 2B, Figure 5B and Figure 8B) demonstrate that gp41rk is deforming the bilayer. The larger $A_{L}$ and smaller headgroup volume of DMPG makes it energetically more favorable for gp41rk to associate with DMPG instead of DMPC because more headgroup-associated water can be displaced from DMPG. This kind of interaction took place in the GP411 and GP414 simulations, but gp41rk in the GP412 simulation was on the hydrocarbon tail side of the phosphate groups. The two peptides in the GP412 simulation created a larger distortion of the bilayer structure, as can be seen in the density profiles of the system (Figure 2A, Figure 5A and Figure 8A), because there is less water in this region to displace. This second cause for lipid-specific interactions is supported by a previous study of gp41rk in a mixture of DMPC, DMPG, POPC and POPG [21] in which the peptide interacted with POPC and POPG preferentially. Both lipids have larger $A_{L}$ values than DMPC and DMPG [46,47].

The differences between the secondary structures seen here and expectations based on experimentally observed results merits discussion. In all three simulations, the DSSP [40,41] results from the simulations do not agree with previous experiments at these concentrations and in this lipid mixture [18,19,21]. Typically, CD results for the HIV-1 fusion peptide are presented as all peptides in the sample having a conformation consistent with the secondary structure fractions observed [14,15,16,17,18,19,20,21,22,23,24,25]. It is also possible that the fractions observed instead reflect ensembles within the population of peptides in the sample. The latter possibility makes the differences observed between the simulations and experiments a matter of sampling. The simulations presented herein represent a very small sampling of trajectory-time space relative to the ensemble that was sampled during the experiments. The SANS measurements involved 300 μL of 5–10 mg/mL lipid concentration with data collection times of hours [18,19,21]. A smaller volume, by roughly a factor of 20, at the same lipid concentration was measured for 10 min for the CD experiments [18,19,21]. Neutron spin echo spectroscopy measurements required an order of magnitude higher sample volume and measurement times as well as concentrations 3 to 6 times higher than the SANS measurements [19,21]. Differences in experimental sample and simulation preparation, which differ greatly for the studies performed by this group [18,19,21], are also quite likely to play a significant role. The experiments began with materials co-dissolved in organic solvent that was blown off and subjected to freeze-drying to ensure that the solvent was removed before resuspension in water and extrusion to make vesicles [18,19,21].

While other molecular dynamics investigations of the HIV-1 FP have been published [52,53,54,55,56], the systems studied did not include a mixture of charged and zwitterionic lipids that would allow for comparisons with the lipid–peptide interactions seen in the current work. Peptides that are classified as membrane-active peptides, which includes peptide toxins and antimicrobial peptides, have been studied in mixed composition bilayers by molecular dynamics simulations. The CM15 peptide, a highly cationic cecropin A-melittin hybrid with antimicrobial activity, was observed to form hydrogen bonds with anionic lipids more frequently than it did with zwitterionic lipids [57]. The peptide also forms salt bridges with anionic lipids [58]. Another antimicrobial peptide, magainin 2, also had stronger interactions with the anionic lipids present in the simulated bilayer than with the zwitterionic ones [59]. However, the same could not be said of the cationic L18W-PGLa peptide investigated in the same study [59]. Melittin, a cationic peptide toxin, displayed affinity for charged lipids in coarse-grained simulations [60]. Collectively, these studies suggest that the driving force behind the preferential association of lipids with these peptides is electrostatic in nature. However, the peptides in these studies [57,58,59,60] were α-helical and adopt a transmembrane conformation that impacts the free energy of the system differently than the head-group-associated state observed here for gp41rk.

The concentration dependence of the interaction of gp41rk with the two lipids in the bilayer is enlightening. Based on the GP411 and GP412 results, the impact of the peptide on the lateral distribution of lipids is largely restricted to the leaflet of the bilayer with which the peptide is associated. These lateral RDFs (Figures S2 and S5) have weak, broad features compared to the lateral RDFs of the peptide-bound leaflet (Figure 4, Figure 7 and Figure 11), as one would expect as a result of the greater distance between the lipids in the opposing leaflet and the peptides’ charged groups. For peptides that are primarily associated with the polar groups of the bilayer, as was seen in the GP411 trajectory and for three of the four peptides in the GP414 trajectory, the enhancement of DMPG in the immediate vicinity of the peptide show signs of concentration dependence, with correlation peaks being clear for distances less than ~10 Å in the GP414 lateral RDFs (Figure 11A,C) that are present but weak in the GP411 curves (Figure 4A). The change suggests a disruption of the gp41rk-DMPG interaction with increasing peptide concentration, although the picture is complicated by the oligomerization observed in the GP414 trajectory for which P/L = 1/50. When the peptides interact significantly with the hydrocarbon core of the bilayer, as was seen in the GP412 results (Figure 7), the charged residues are correlated with the charged phosphate group of the lipids regardless of the net charge on the lipid. However, the polar and hydrophobic residues of the peptide continue to preferentially associate with DMPG over DMPC. Regardless of the kind of amino acid residue type, there is clearly more lateral structure induced at P/L = 1/100 (GP412) than exists at P/L = 1/200 (GP411). The increased lateral ordering may be part of the driving force for the conformational change of gp41rk in model lipid bilayers made solely of a mixture of charged and zwitterionic lipids, but this cannot be said conclusively because the present work did not observe the helix-to-sheet transition that was seen experimentally [18,19,21].

## 5. Conclusions

The simulations performed here provide new insight into the lipid-specific interactions of gp41rk with lipid bilayer membranes shortly after the peptide binds to the membrane. The results reveal preferential association of charged lipids with the gp41rk peptides associated with the bilayer. The preferential interactions of gp41rk with charged lipids seen at low concentrations remains as the peptide concentration increases. The vicinity of the peptide is enhanced with charged lipids at the expense of zwitterionic ones even when the peptide oligomerizes. The preferential association of gp41rk with DMPG is also maintained even when the peptide penetrates into the hydrocarbon core of the bilayer. The results indicate that lipid-specific interaction is an important aspect of the interaction of the peptide with the bilayer.

## Figures and Tables

**Table 1:** The systems simulated, abbreviations used, and total length of time simulated. The ‘*’ denotes that the total time of the production simulation is a combination of the production run performed during the previous study (200 ns) [21] and an additional 800 ns of production simulation time performed for the present study.

System	Abbreviation	Total Production Time (ns)
7:3 DMPC:DMPG, no peptide	GP410	1000 *
7:3 DMPC:DMPG, 1 gp41rk	GP411	1000
7:3 DMPC:DMPG, 2 gp41rk	GP412	1000
7:3 DMPC:DMPG, 4 gp41rk	GP414	2000

## Data Availability

Data are available from the author on request.

## Supplementary Materials

The following supporting information can be downloaded at: https://www.mdpi.com/article/10.3390/biom14030285/s1, Figure S1: Number of contacts and minimum distance between the peptide and lipids in the GP411 simulation; Figure S2: Lateral RDFs between the peptides and the lipid P atoms in the GP411 simulation for the leaflet of the bilayer that the peptide is not bound to; Figure S3: Number of contacts and minimum distance between the peptides and the lipids in the GP412 simulation; Figure S4: Number of contacts and minimum distance between the peptides in the GP412 simulation; Figure S5: Lateral RDFs between the peptides and the lipid P atoms in the GP412 simulation for the leaflet of the bilayer that the peptide is not bound to; Figure S6: Number of contacts and minimum distance between the peptides and the lipids in the GP414 simulation; Figure S7: Number of contacts and minimum distance between the pairs of peptides in each leaflet of the bilayer in the GP414 simulation.

## Abbreviations

The following abbreviations are used in this manuscript:

FP	fusion peptide
P/L	peptide-to-lipid molar ratio
GP410	simulation of 7:3 DMPC:DMPG with no peptide
GP411	simulation of 7:3 DMPC:DMPG with one gp41rk
GP412	simulation of 7:3 DMPC:DMPG with two gp41rk
GP414	simulation of 7:3 DMPC:DMPG with four gp41rk
SANS	Small-angle neutron scattering
NSE	Neutron spin echo spectroscopy
CD	Circular dichroism
MD	Molecular dynamics
PME	Particle mesh Ewald
RDF	Lateral radial density function
$D_{ptp}$	Phosphate-to-phosphate distance
DMPC	1,2-dimyristoyl-sn-glycero-3-phosphocholine
DMPG	1,2-dimyristoyl-sn-glycero-3-phospho-(1’-rac-glycerol) sodium salt
POPC	1-palmityol-2-oleoyl-sn-glycero-3-phosphocholine
POPG	1-palmityol-2-oleoyl-sn-glycero-3-phospho-(1’-rac-glycerol) sodium salt
POPS	1-palmitoyl-2-oleoyl-sn-glycero-3-phospho-L-serine sodium salt
