# Supplementary material for: Molecular Dynamics Investigation of Lipid-Specific Interactions with a Fusion Peptide
Source: Biomolecules. 2024 Feb 27;14(3):285. doi: 10.3390/biom14030285 (PMC10968095; doi:10.3390/biom14030285)

Supporting Information for:

Molecular Dynamics Investigation of Lipid-Specific Interactions with a Fusion Peptide

William T. Heller[1]

[1]Neutron Scattering Division, Oak Ridge National Laboratory, Oak Ridge, TN 37831, United States of America

Notice: This manuscript has been authored by UT-Battelle, LLC, under contract DE-AC05-00OR22725 with the US Department of Energy (DOE). The US government retains and the publisher, by accepting the article for publication, acknowledges that the US government retains a nonexclusive, paid-up, irrevocable, worldwide license to publish or reproduce the published form of this manuscript, or allow others to do so, for US government purposes. DOE will provide public access to these results of federally sponsored research in accordance with the DOE Public Access Plan (http://energy.gov/downloads/doe-public-access-plan).

Figure S1. (A) Number of contacts between the gp41rk peptide in the GP411 simulation with lipid molecules as a function of time. (B) Minimum distance between the gp41rk peptide in the GP411 simulation and an atom of a lipid molecule as a function of time.

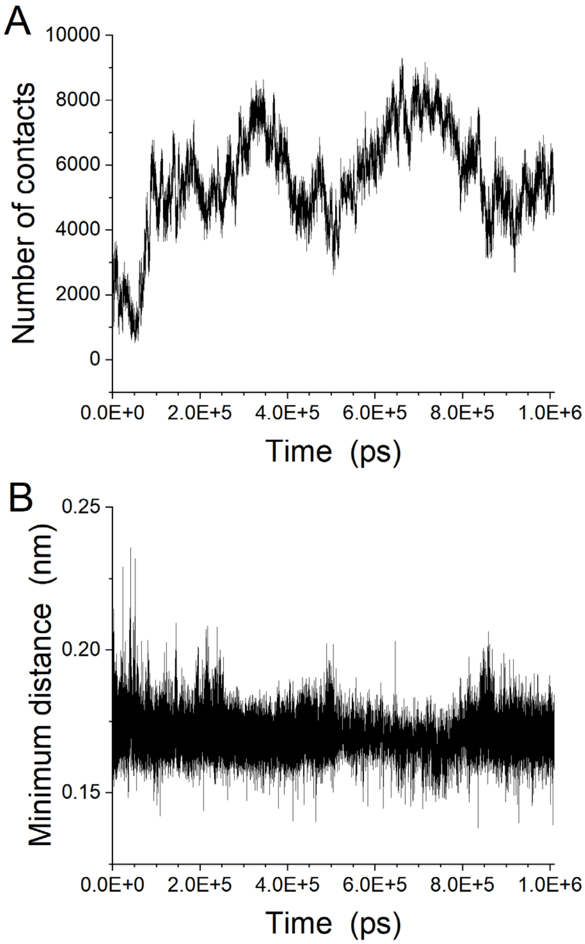

Figure S2. Peptide-Lipid RDFs from the GP411 simulation. (A) radial distribution functions between DMPC P atoms in the leaflet of the bilayer to which the peptide is ***not*** bound and gp41rk hydrophobic residues (black), polar residues (red) and positive residues (blue). (A) radial distribution functions between DMPG P atoms in the leaflet of the bilayer to which the peptide is ***not*** bound and gp41rk hydrophobic residues (black), polar residues (red) and positive residues (blue).

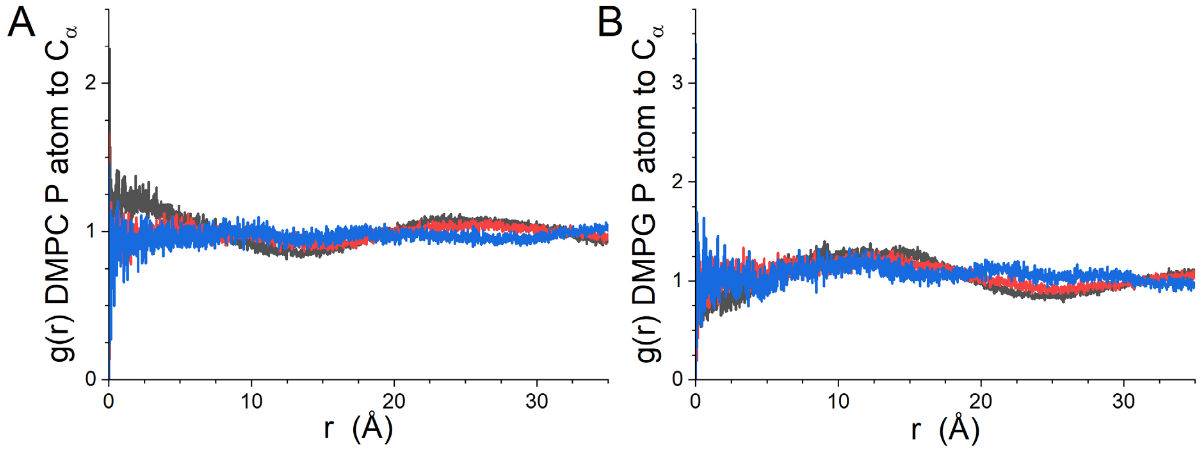

Figure S3. (A) Number of contacts between the gp41rk peptide #1 (black) and the gp41rk pepide #2 (red) in the GP412 simulation with lipid molecules as a function of time. (B) Minimum distance between the gp41rk peptide #1 (black) and the gp41rk peptide #2 (red) in the GP412 simulation and an atom of a lipid molecule as a function of time.

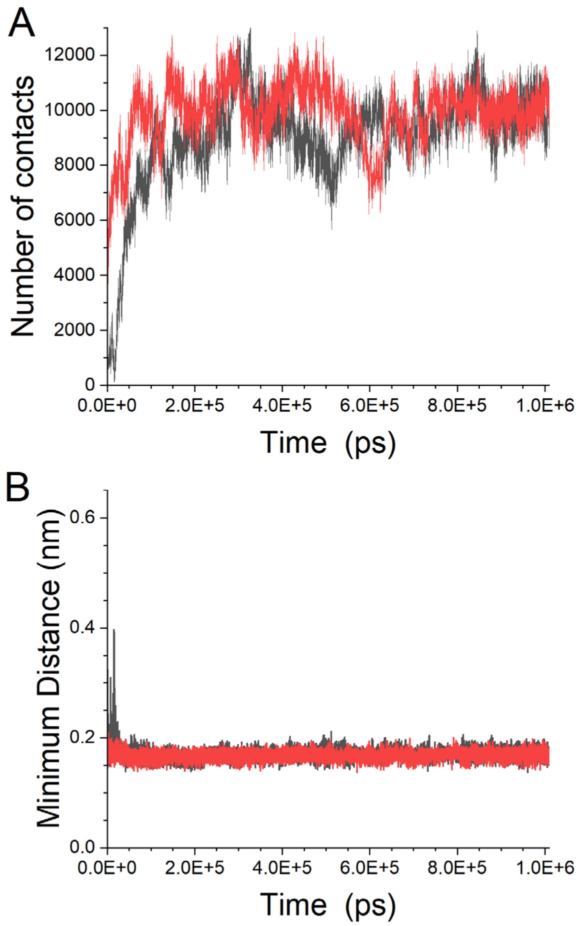

Figure S4. (A) Number of protein-protein contacts in the GP412 simulation as a function of time and (B) minimum distance between the proteins as a function of time.

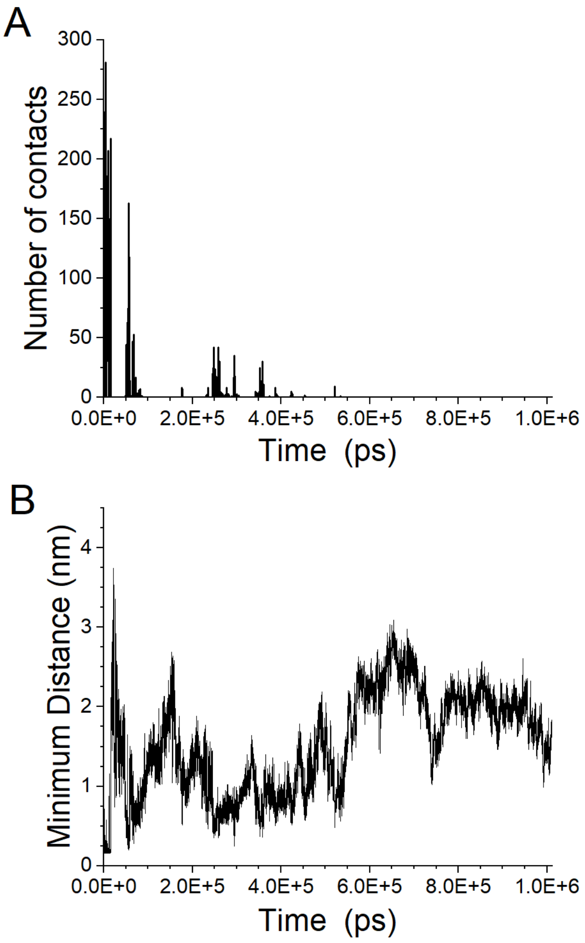

Figure S5. Peptide-Lipid RDFs from the GP412 simulation. (A) radial distribution functions between DMPC P atoms in the leaflet of the bilayer to which the peptides are ***not*** bound and gp41rk hydrophobic residues (black), polar residues (red) and positive residues (blue). (A) radial distribution functions between DMPG P atoms in the leaflet of the bilayer to which the peptides are ***not*** bound and gp41rk hydrophobic residues (black), polar residues (red) and positive residues (blue).

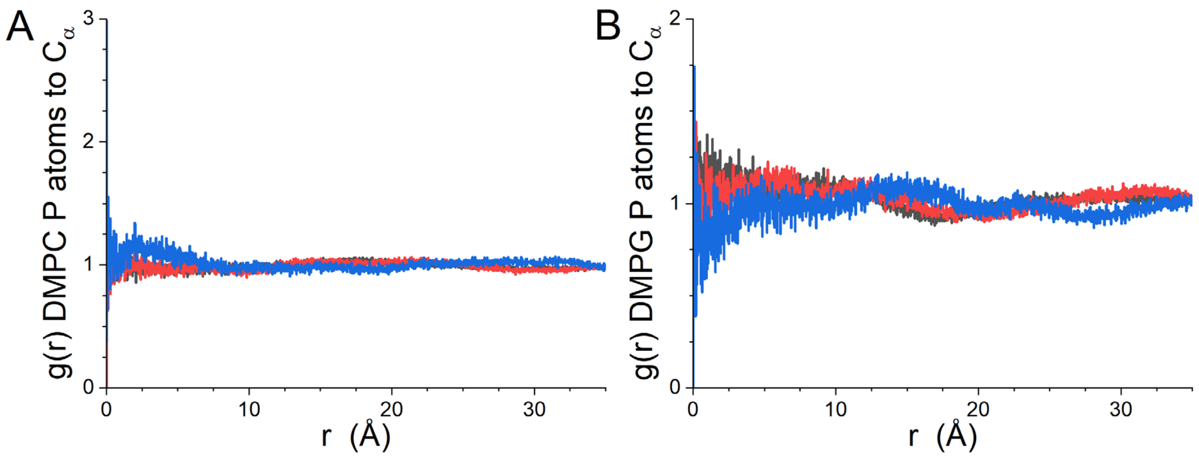

Figure S6. (A) Number of contacts between the gp41rk peptide #1 (black), gp41rk peptide #2 (red), gp41rk peptide #3 (blue) and gp41rk peptide #4 (green) in the GP414 simulation with lipid molecules as a function of time. (B) Minimum distance between the gp41rk gp41rk peptide #1 (black), gp41rk peptide #2 (red), gp41rk peptide #3 (blue) and gp41rk peptide #4 (green) in the GP414 simulation and an atom of a lipid molecule as a function of time.

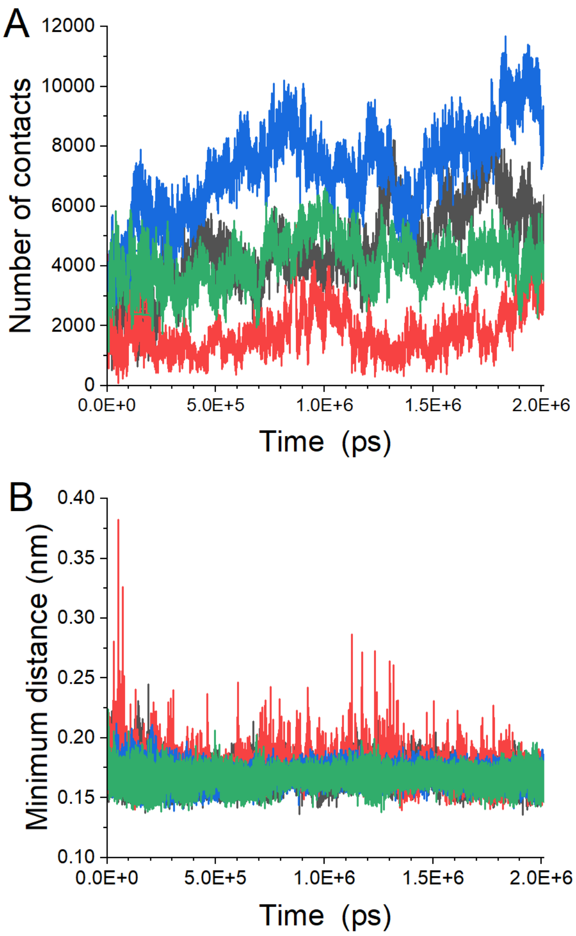

Figure S7. (A) Number of protein-protein contacts in the GP414 simulation as a function of time for the gp41rk #1 - #4 pair (black) and the gp41rk #2 - #3 pair (red) and (B) minimum distance between the gp41rk #1 - #4 pair (black) and the gp41rk #2 - #3 pair (red) as a function of time.

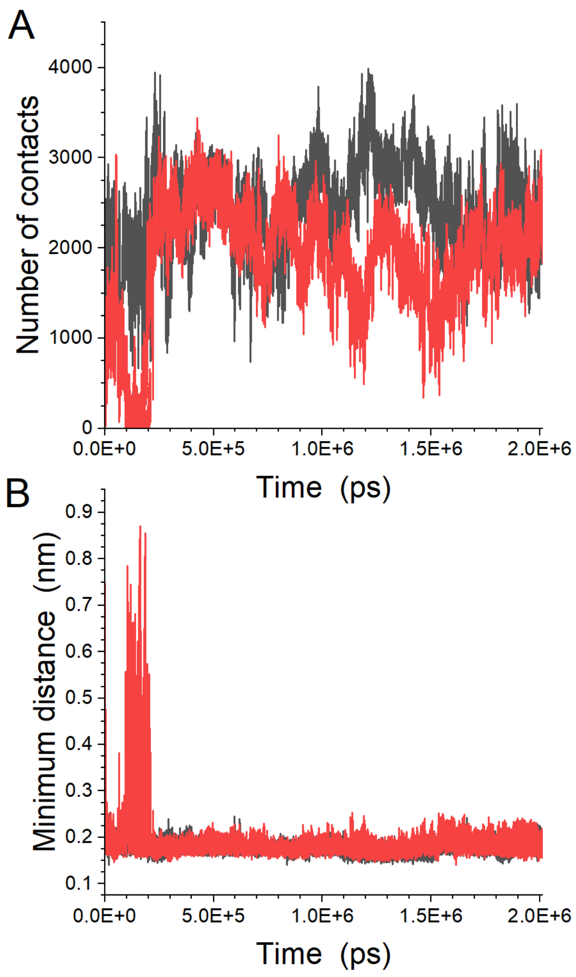

Supplement: Supplementary file 1 [file biomolecules-14-00285-s001.zip › lipid_peptide11v2_si.pdf]
